# Feeding a nutrient enriched diet to late gestating sows across consecutive cycles improves micronutrient status, farrowing duration, and prolificacy

**DOI:** 10.1093/tas/txaf155

**Published:** 2025-11-24

**Authors:** Katlyn A McClellan, Jesus Acosta, Brad Lawrence, Sara Hough, Jon Bergstrom, Mark Weaver, Rebecca Robbins, Eric M Weaver

**Affiliations:** Department of Animal Science, South Dakota State University, Brookings, SD, 57007, United States; Novus International, Inc, Chesterfield, MO, 63005, United States; Novus International, Inc, Chesterfield, MO, 63005, United States; dsm-firmenich, Plainsboro, NJ, 08536, United States; dsm-firmenich, Plainsboro, NJ, 08536, United States; Pig Improvement Company, Hendersonville, TN, 37075, United States; Pig Improvement Company, Hendersonville, TN, 37075, United States; Department of Animal Science, South Dakota State University, Brookings, SD, 57007, United States

**Keywords:** gestation, phase feeding, piglet, nutrition, sow

## Abstract

This study evaluated effects of a nutrient-enriched late gestation diet (LGPHASE) on reproductive performance and blood-based biomarkers in sows and their progeny through weaning. Seventy sows (parity 0–5) at 70 ± 2 d gestation were assigned to a control (CON; *n* = 35; 11% CP, 0.52% SID lysine, industry-standard organic trace minerals, and vitamins) or a LGPHASE diet (*n* = 35; 16% CP, 0.87% SID lysine, 2 × CON industry-standard organic trace minerals and vitamins except selenium, plus 500 mg/kg vitamin C) fed from days 70 to 110 of gestation. All sows received a common lactation diet. Blood was collected from sows at day 70 ± 2, day 110 ± 2, and weaning (lactation day 19 ± 3) for blood counts and serum analysis. Piglet Hb was measured at birth (CON: *n* = 479; LGPHASE: *n* = 495) and at weaning (CON: *n* = 395; LGPHASE: *n* = 431), and serum was collected from 2 piglets per litter (*n* = 70 per treatment) at 40–48 h post farrow and weaning. Sow and piglet serum was analyzed for copper, iron, zinc, ferritin, and 25(OH)D_3_. Farrowing duration was shorter in LGPHASE than CON sows (290.4 vs. 359.9 min; *P *= 0.028). Litter size and birth weight were similar between treatments, however, a reduced stillbirth rate tended to occur in LGPHASE sows (3.4% vs. 6.8%; *P *= 0.099). LGPHASE sows tended to wean more (13.4 vs. 12.3; *P *= 0.062) and lighter pigs (5.70 vs. 6.16 kg; *P *= 0.087) with increased piglet survivability (90.5% vs. 84.8%; *P *= 0.019). LGPHASE sows tended to have higher Hb at day 110 (11.2 vs. 10.7 g/dL; *P *= 0.091) and had higher Hb at weaning (10.7 vs. 9.9 g/dL; *P *= 0.004) than CON sows. At day 110, LGPHASE sows had greater serum 25(OH)D_3_ (54.8 vs. 35.5 ng/mL; *P *= 0.002), and tended to have greater serum ferritin at weaning than CON sows (38.5 vs. 28.3 ng/mL; *P *= 0.074). At day 2, LGPHASE piglets had greater serum ferritin (25.1 vs. 19.1 ng/mL; *P *= 0.079). At weaning, serum copper was greater in LGPHASE piglets (2.26 vs. 2.17 µg/mL; *P *= 0.022) and serum 25(OH)D_3_ tended to be greater in the LGPHASE piglets than CON piglets (7.5 vs. 6.9 ng/mL; *P *= 0.080). In the subsequent cycle, LGPHASE sows had more total born (19.24 vs. 16.84; *P *= 0.041) and live born pigs (17.70 vs. 15.00; *P *= 0.015), reflecting increases of 2.74 total born (*P *= 0.024) and 2.50 live born pigs (*P *= 0.031) from the prior cycle. Late-gestation nutrient needs exceed typical feeding levels; phase feeding improved micronutrient status, farrowing duration, piglet survival, and subsequent litter size.

## Teaser

A nutrient-enriched late gestation diet fed to prolific sows shortened farrowing duration, lowered pre-weaning mortality, and improved maternal and neonatal micronutrient status.

## Introduction

Genetic progress over the past decade has markedly increased sow prolificacy, with many herds now averaging around 16 total born per parity. Some hyper-prolific sows are farrowing more than 20 (Johannsen et al. 2024; PigCHAMP 2025). These large litters places substantial metabolic and subsequently nutritional demands on the sow, particularly in the last trimester when fetal growth and mammary development peak (McPherson et al. 2004; Theil et al. 2022). To meet these heightened demands, late-gestation diets must provide adequate daily nutrient intake to support both fetal development, mammary growth and maternal growth in young sows. Yet, traditional feeding strategies may fall short which may be one of the contributing causes of high sow removal rates (Monteiro et al. 2025).

After day 70 of gestation, fetal weight increases rapidly (McPherson et al. 2004). Mammary development accelerates to prepare for lactation (Kim et al. 2009), and maternal blood volume expands to sustain uteroplacental growth and fetal oxygen transport (Anderson et al. 1970; Matte and Girard 1996). Previous research has demonstrated a substantial rise in lysine requirements as gestation progresses. Samuel et al. (2012) reported that total lysine requirements nearly doubled, from 9.4 g/d in early gestation to 17.4 g/d in late gestation. These findings highlight the importance of adjusting dietary formulations to meet the sow’s increasing nutrient demands during pregnancy. However, current gestation lysine recommendations would require late gestation intakes of approximately 2.7 kg/d (NRC 2012). Assuming a common gestation diet fed to the entire herd, the elevated caloric intake associated with prolonged intake at these levels has produced variable responses in the literature and has been linked in some cases to poorer farrowing outcomes (Gonçalves et al. 2016). Although our understanding of amino acid nutrition has advanced, most studies evaluating protein requirements in late gestation were conducted with sows of lower reproductive potential than today’s prolific genotypes (Jo and Kim 2023; Samuel et al. 2012; Shi et al. 2016).

Data on sow demands for available trace minerals and vitamins remain limited, particularly for prolific sows. Although the NRC (2012) notes that current genotypes may have increased micronutrient requirements, the requirement estimates have remained unchanged due to the limited availability of research specifically designed to support revisions to these values.

Mahan and Newton (1995) indicated that sow mineral reserves are progressively depleted over three parities, with higher-producing sows experiencing greater losses of both macro- and microminerals than their less productive counterparts. To address the presumed increase in micronutrient needs in modern high-producing sows, the industry has adopted higher dietary fortification levels of trace minerals and vitamins compared to estimated requirements (Faccin et al. 2023; NRC 2012). However, this practice is primarily driven by practical considerations rather than empirical evidence, highlighting the lack of definitive data on trace mineral requirements in prolific sows.

Maternal under-nutrition impacts both the dam and the offspring. Anemia stands out as a significant health issue in pigs affected by maternal nutrition. Castevens et al. (2020) reported that nearly 50% of gestating sows were anemic (hemoglobin < 10 g/dL whole blood; Bhattarai et al. 2018). Although anemia generally reflects low iron status (Underwood and Suttle 1999), its prevalence and underlying causes in sows remain poorly understood and are likely influenced by multiple nutritional factors. Iron status is shaped not only by dietary iron intake but also by the availability of other nutrients essential for iron absorption, transport, and storage as well as blood volume necessary to support the developing litter. Nutrients interact closely with deficiencies or excesses in one potentially impairing the availability or utilization of others as a result of shared transporters, coenzyme functions, or protein synthesis pathways (Goff 2018). For instance, vitamin C enhances intestinal iron absorption by maintaining iron in its bioavailable ferrous form (Lane and Richardson 2014), whereas vitamins A and D influence iron mobilization and mineral homeostasis via effects on transferrin receptor expression and endocrine regulation (Michelazzo et al. 2013; Smith and Tangpricha 2015). Inadequate protein intake can also limit the synthesis of iron-binding proteins and enzymes critical for mineral metabolism, including transferrin, ferritin, and ceruloplasmin (Underwood and Suttle 1999). These interactions suggest that simply increasing dietary iron may be insufficient to correct anemia if other nutrient deficiencies are present—particularly during late gestation, when the requirements for amino acids, vitamins, and trace minerals are concurrently elevated.

Although nutrient requirements are known to increase throughout gestation, limited research has evaluated multi-nutrient phase-feeding strategies that simultaneously elevate amino acid, trace mineral, calcium, phosphorus, and vitamin concentrations. The effects of such strategies on hematological and nutritional biomarkers in sows and their offspring remain poorly understood. This study aimed to assess the impact of a nutrient-enriched, phase-fed diet during late gestation on farrowing duration, maternal and progeny nutritional biomarkers, and reproductive performance.

## Materials and methods

### Animal care and use

All procedures used in this study were approved by the South Dakota State University (SDSU) Institutional Animal Care and Use Committee. Animals used in this experiment were raised and managed in the sow barn at SDSU Swine Education and Research Facility, located in Brookings, South Dakota.

### Animals used and housing

A total of 70 female pigs (PIC Camborough 42; parities 0 to 5) and their offspring (*n* = 1051 piglets) were used in this experiment, which spanned from late gestation (70 ± 2 d of gestation) through weaning (19 ± 3 d of lactation). During gestation, sows were housed in group pens until day 110 ± 2 of gestation, after which they were moved to individual farrowing crates until weaning.

### Dietary treatments and feeding

Females were allocated to one of two dietary treatments, balanced by parity, body weight (BW), initial blood hemoglobin concentration (Hb), and prior reproductive performance (total born, liveborn, and pigs weaned. Gilts were excluded from this allocation factor). The females received either a control (CON; *n* = 35; 11% CP, 0.52% SID lysine, industry-standard organic trace minerals, and vitamins) or a LGPHASE (*n* = 35; 16% CP, 0.87% SID lysine, 2 × CON levels of organic trace minerals and vitamins, except selenium, plus 500 mg/kg vitamin C) diet (Table 1). Dietary treatments were fed from 70 ± 2 d of gestation to 110 ± 2 d of gestation. Sows that remained in the herd for a subsequent reproductive cycle (CON: 21 sows; LGPHASE: 28 sows) were re-enrolled on their originally assigned diets at day 70 of their subsequent gestation. Daily feed allowances during gestation were determined for each sow to meet or exceed nutrient and energy requirements for maintenance, parity-appropriate maternal growth, and fetal and mammary development (NRC 2012). These allowances were further adjusted based on body condition score assessment (sows were categorized as thin, ideal, or over-conditioned).

**Table 1.** Composition of gestation and lactation diets (as-fed basis).

Item^a^	Gestation		Lactation
	CON	LGPHASE	
**Ingredient, %**			
** Ground corn**	84.53	68.30	66.21
** Soybean meal, 46.5% CP**	7.67	22.16	29.85
** Soybean hulls**	5.00	5.00	0.00
** Limestone**	0.70	1.01	1.22
** Monocalcium phosphate, 21%**	0.99	1.32	1.76
** Soybean oil**	0.00	0.47	0.00
** Salt**	0.50	0.50	0.50
** Trace mineral premix^b^**	0.25	0.50	0.15
** L-lysine, HCL 99%**	0.19	0.20	0.00
** Vitamin C^c^**	0.00	0.14	0.00
** Vitamin premix^d^**	0.05	0.10	0.05
** L-threonine**	0.04	0.12	0.00
** DL-methionine**	0.00	0.10	0.00
** Phytase^e^**	0.08	0.08	0.03
** Swine toxin binder^f^**	0.00	0.00	0.10
** Larvicide^g^**	0.00	0.00	0.13
**Calculated composition**			
** Metabolizable energy, kcal/kg**	3185	3205	3226
** Crude protein, %**	11.04	16.59	19.52
** Calcium, %**	0.61	0.83	0.94
** Total phosphorus, %**	0.49	0.62	0.75
** Phosphorus, avail. %**	0.46	0.54	0.54
**Amino acids, SID basis, %**			
** Lysine**	0.53	0.87	0.97
** Threonine**	0.37	0.62	0.62
** Methionine**	0.18	0.33	0.28
** Methionine + cysteine**	0.36	0.65	0.54
** Tryptophan**	0.09	0.16	0.20
** Isoleucine**	0.36	0.60	0.72
** Valine**	0.45	0.69	0.80
** Arginine**	0.28	0.47	1.17
** Histidine**	0.18	0.30	0.47
** Leucine**	1.02	1.33	1.55
** Phenylalanine + tyrosine**	0.53	0.87	0.85
**Analyzed composition**			
** Crude protein, %**	10.89	16.53	18.99
** Crude fat, %**	2.29	2.28	2.22
** Crude fiber, %**	3.23	3.11	2.07
** Ash, %**	3.80	4.68	5.48
** Copper (SD) mg/kg^h^**	24.9 (4.6)	32.4 (4.4)	19.3 (1.1)
** Iron (SD) mg/kg^h^**	195.5 (19.1)	313.3 (27.7)	282.0 (16.7)
** Manganese (SD) mg/kg^h^**	64.2 (17.0)	120.2 (17.8)	77.6 (5.3)
** Zinc (SD) mg/kg^h^**	168.0 (15.6)	316.4 (15.0)	105.0 (3.4)
** Vitamin A (SD) IU/kg^h^**	10,180 (2820.3)	17,150 (3709.4)	9078 (2342.7)
** Vitamin C (SD) mg/kg^h^**	…	460.0 (40.0)	…
** Vitamin D_3_ (SD) IU/kg^h^**	2170.5 (305.1)	3883.0 (944.2)	2140.6 (488.2)
** Vitamin E (SD) IU/kg^h^**	94.6 (14.7)	184.8 (37.4)	110 (24.6)

a Experimental gestation diets were fed from day 70 of gestation to day 110 of gestation, and common lactation diet was fed from day 110 until weaning in each cycle.

b Minimum levels provided per kg of diet: Gestation (CON): Copper (15 mg, methionine hydroxy analogue chelate, Novus International Inc.); Iodine (0.21 mg, J & R Distributing Inc); Iron (118 mg, polysaccharide complexed, QualiTech LLC); Manganese (37 mg, methionine hydroxy analogue chelate, Novus International Inc.); Selenium Yeast (0.3 mg, Alltech); Zinc (148 mg, methionine hydroxy analogue chelate, Novus International Inc.).

Gestation (LGPHASE): Copper (29.6 mg, methionine hydroxy analogue chelate, Novus International Inc.); Iodine (0.42 mg, J & R Distributing Inc); Iron (236 mg, polysaccharide complexed, QualiTech LLC); Manganese (74 mg, methionine hydroxy analogue chelate, Novus International Inc.); Selenium Yeast (0.3 mg, Alltech); Zinc (296 mg, methionine hydroxy analogue chelate, Novus International Inc.), Lactation: Copper (15 mg, J & R Distributing Inc); Iodine (0.36 mg, J & R Distributing Inc); Iron (125 mg, J & R Distributing Inc); Manganese (40 mg, J & R Distributing Inc); Selenium Yeast (0.3 mg, J & R Distributing Inc); Zinc (156 mg, J & R Distributing Inc).

c Vitamin C (Stay C 35, DSM Nutritional Products Inc.).

d Minimum levels provided the following per kilogram of diets: Vitamin A 11,000 IU, Vitamin D3 1650 IU, Vitamin E 55 IU; Vitamin B12 0.044 mg, Menadione 4.4 mg, Biotin 0.165 mg, Folic Acid 1.1 mg, Niacin 55 mg, d-Pantothenic Acid 60.5 mg, Vitamin B6 6.3 mg, Riboflavin 9.9 mg, Thiamin 3.3 mg (J & R Distributing Inc).

e Commercial feed-grade phytase (Quantum Blue, AB Vista, Marlborough, UK) at 2000 FTU/kg.

f Algonite; blend of dried yeast cells, diatomaceous earth, and algae (Olmix NA Inc.).

g Minimum levels provided the following per kilogram of diets: Active Ingredient: Tetrachlorovinphos 75.9 mg (Elanco US Inc.).

h Represents the mean of five subsamples analyzed within a given diet.

Individual daily feed allotments were delivered via electronic sow feeders (Gestal 3G, Jyga Technologies Inc.). At 110 ± 2 d of gestation, sows were moved to farrowing crates and transitioned to a common lactation diet formulated to meet or exceed NRC (2012) requirements for lactating gilts. From day 110 until farrowing, feed intake was increased to 2.7 kg/d and delivered in six meals between 5:00 AM and 8:00 PM using an automated feeding system (Gestal Solo, Jyga Technologies Inc.). After farrowing, sows were provided ad libitum access to feed for the remainder of lactation, with daily intake estimates recorded.

### Diet sample collection and analysis

Representative samples of each dietary treatment, as well as the common lactation diet, were collected throughout the feeding period and pooled by treatment. Five composite samples per diet were analyzed, and the average values were used to represent each diet’s nutrient composition, including trace minerals and vitamins. Proximate composition—comprising dry matter, crude protein, ether extract, crude fiber, crude fat, and ash—was determined using official methods of the Association of Official Analytical Chemists (AOAC) (AOAC International 2023). Trace minerals (copper, iron, manganese, and zinc) were quantified by inductively coupled plasma–optical emission spectrometry, while selected vitamin concentrations (A, D, E, and C) were measured using modified AOAC methods with validated chromatographic and spectrophotometric techniques.

### Sow sampling and processing

Throughout Cycle I of the experiment, females were weighed and blood samples were collected on day 70 (± 2) of gestation (prior to diet al. ocation), day 110 (± 2) of gestation, and days 2 and 19 (± 3) of lactation. Additionally, sows had last rib backfat (BF) measured via ultrasonography on day 70 (± 2) and day 110 (± 2) of gestation, as well as day 19 (± 3) of lactation. For blood collection, sows were restrained using a snare, and samples were obtained from the jugular vein using 18-gauge × 1½ in. aluminum hub needles (Animal Health International, Loveland, CO). Blood was collected into two types of tubes: 6 mL EDTA tubes (Greiner Bio-One VACUETTE™ K3EDTA Blood Collection Tubes; Fisher Scientific, Waltham, MA) for whole blood analysis, and 6 mL plain serum tubes (BD Vacutainer™ Plus Plastic Serum Tubes, Silicone-Coated with Hemogard™ Closure; Fisher Scientific, Waltham, MA) for serum separation.

### Reproductive performance data

Farrowing was monitored using surveillance cameras (Reolink, New Castle, DE) and a 24-h farrowing supervision protocol, with trained research personnel conducting farrowing watch in rotating shifts. Manual internal examination (sleeving) was conducted to assess for the presence of a piglet in the birth canal when 60 min had elapsed since the birth of the previous piglet. To maintain hygiene, obstetrical sleeves and lubricant were used, and care was taken to avoid contact between gloved hands and crate surfaces prior to sleeving. If a piglet was palpated in the birth canal during sleeving, it was manually extracted. Only one piglet was retrieved per sleeve unless multiple piglets were present in the birth canal simultaneously, in which case all were retrieved. Subsequent sleeving was delayed until another 60 min had passed without piglet expulsion. Farrowing duration was defined as the time elapsed between the birth of the first and last piglet in each litter. Stillborn piglets were identified using the lung flotation method (Bhattarai et al. 2018). Cross-fostering was limited to within treatment groups, and piglets were only redistributed to align litter size with the functional teat capacity of the sow. No piglets were moved between treatments.

### Piglet sampling

All piglets were individually weighed within 6 h of birth and again at weaning. Blood Hb concentration was measured within 6 h of birth (CON: *n* = 479; LGPHASE: *n* = 495) and at weaning (CON: *n* = 395; LGPHASE: *n* = 431) for all live piglets, using a point-of-care device to minimize total blood volume collected. For this, the ear vein was punctured using a 20-gauge × 1 in. needle, and a droplet of blood was collected into disposable microcuvettes via capillary action. The Hb concentration was measured immediately using a HemoCue Hb 201+ analyzer (HemoCue America, Brea, CA), with results displayed and recorded within 60 s. The accuracy of this device was previously validated in this population, demonstrating <1% deviation from laboratory analysis of ear vein samples (McClellan et al. 2024b). Additionally, jugular blood samples were collected from two average-weight piglets per litter (one male and one female; *n* = 70 per treatment) between 20 and 24 h after birth, prior to iron administration. At weaning, two average-weight piglets per litter (one male and one female; *n* = 70 per treatment) were again selected and sampled. Jugular blood was collected using 21-gauge × ½ in. aluminum hub needles (Animal Health International, Loveland, CO) into 6 mL plain serum tubes (BD Vacutainer™ Plus Plastic Serum Tubes, silicone-coated with Hemogard™ Closure; Fisher Scientific, Waltham, MA).

One of the largest piglets from each of 15 liters per treatment group (*n* = 30) was removed within 36 h of birth for a parallel tissue development study not described herein. These piglets were included in total born, liveborn, and birth weight analyses but were censored at the time of removal and therefore excluded from all postnatal survival calculations. Piglet survivability was defined as the proportion of liveborn piglets that remained alive at weaning.

### Whole blood sample handing and analysis

The EDTA-treated whole blood samples from sows were gently mixed immediately after collection and maintained at room temperature until analysis. Samples were analyzed within 6 ± 2 h of collection to assess hematological parameters, including Hb concentration, red blood cell (RBC) count, white blood cell (WBC) count, packed cell volume (PCV), and platelet (PLT) count. All analyses were conducted using the Siemens Advia 2120/2120i Hematology System (Siemens Healthcare Diagnostics, Eschborn, Germany).

### Serum sample handling and processing

Blood samples for serum analyses were set to clot at room temperature for 1 h following collection and then centrifuged at 2000 × g for 20 min. Serum was separated and stored at −20°C until analysis.

### Serum ferritin quantification

Serum ferritin concentrations were measured using a quantitative immunoturbidimetric assay on the Vet AXCEL Clinical Chemistry System (Alfa Wassermann, West Caldwell, NJ, USA). The assay quantifies ferritin based on turbidity generated by antigen-antibody complexes formed between serum ferritin and specific anti-ferritin antibodies, with absorbance measured photometrically. Assay calibration and quality control were performed according to the manufacturer’s specifications, and intra-assay coefficients of variation were maintained below 10%.

### Serum trace mineral analysis (copper, iron, zinc)

Serum mineral concentrations were determined using inductively coupled plasma–optical emission spectrometry following microwave-assisted nitric acid digestion. Approximately 0.5 g of serum was digested in concentrated nitric acid within a closed-vessel microwave digestion system (220 °C, 20-min hold). After digestion, samples were diluted to 50 mL with deionized water and analyzed for copper, iron, and zinc. Calibration standards and certified reference materials were employed to ensure analytical accuracy, and internal standards were included to correct for instrument drift and matrix effects. Quality control criteria required recoveries within ±10% of target values.

### Serum vitamin D metabolite analysis

Serum concentrations of 25(OH)D_3_ were quantified using liquid chromatography–tandem mass spectrometry. Samples underwent protein precipitation with 0.2 M zinc sulfate solution, followed by extraction with methanol and hexane. Chromatographic separation was performed using an Agilent 1290 Infinity HPLC system coupled to an Agilent 6460 triple quadrupole mass spectrometer equipped with an electrospray ionization source. Calibration was established using National Institute of Standards and Technology reference materials. The assay demonstrated analytical accuracy greater than 95%, with intra- and inter-assay coefficients of variation below 5%.

### Statistical analysis

All data were analyzed using SAS version 9.4 (SAS Institute Inc., Cary, NC). Assumptions of normality and homogeneity of variances were assessed to ensure the validity of the ANOVA-based models. Continuous variables were analyzed using the MIXED procedure, with dietary treatment included as a fixed effect. Total pigs born was included as a covariate for the analysis of farrowing duration. Number of pigs weaned was included as a covariate for the analysis of piglet wean weight and total litter wean weight. Treatment means were compared using Tukey’s honest significant difference test. Categorical data such as sow removal rates were summarized descriptively due to limited sample size and were not subjected to inferential statistical testing. Reproductive performance outcomes across two consecutive cycles for sows that remained in the herd for the subsequent parity were analyzed using a repeated-measures mixed model with PROC GLIMMIX. Treatment, cycle, and their interaction were included as fixed effects, and sow ID was included as a random effect to account for repeated measurements. Least squares means were reported, and significance was declared at *P *≤ 0.05, with trends noted at 0.05 < *P *≤ 0.10.

## Results

### Sow body weight, backfat, and feed intake

Sow BW did not differ between dietary treatments at any timepoint, nor did BW change during gestation (70 ± 2 to 110 ± 2 d of gestation), lactation (day 2 to 19 ± 3 d of lactation), or overall (70 ± 2 d of gestation to 19 ± 3 d of lactation) (Table 2).

**Table 2.** Effects of a late-gestation phase-feeding program on sow body weight (BW), backfat (BF), and average daily feed intake (ADFI) in cycle I.

Item	Dietary treatment		SEM	*P*-value
	CON	LGPHASE		
**Sows, n**	35	35	…	…
**Avg. sow parity, n^a^**	1.6	1.6	…	…
**Sow BW, kg**				
** day 70 gestation**	213.8	214.9	4.03	0.842
** day 110 gestation**	249.7	250.3	4.98	0.915
** day 2 lactation**	242.4	239.7	5.65	0.638
** day 19 lactation**	239.7	242.8	5.63	0.577
**Gestation BW change, kg^b^**	35.9	35.4	3.33	0.874
**Lactation BW change, kg^c^**	−2.7	3.1	2.68	0.126
**Overall BW change, kg^d^**	25.9	27.9	3.74	0.603
**Sow backfat, mm**				
** day 70 gestation**	11.73	11.18	0.37	0.312
** day 110 gestation**	11.84	12.75	0.43	0.098
** day 19 lactation**	11.21	11.67	0.44	0.413
**Gestation BF change, mm^b^**	0.11	1.57	0.42	0.020
**Lactation BF change, mm^c^**	−0.63	−1.08	0.51	0.095
**Overall BF change, mm^e^**	−0.52	0.49	0.38	0.074
**Gestation ADFI, kg/d**	2.17	2.17	0.01	0.943
**Lactation ADFI, kg/d**	7.23	7.53	0.26	0.283

a Parity distribution (CON vs. LGPHASE): Parity 0 = 8 vs. 8; Parity 1 = 14 vs. 14; Parity 2 = 6 vs. 6; Parity 3 = 2 vs. 2; Parity 4 = 1 vs. 1; Parity 5 = 4 vs. 4.

b Differences refer to values on day 110 vs. day 70 of gestation.

c Differences refer to values on day 2 vs. day 19 of lactation.

d Differences refer to values on 70 of gestation vs. day 19 of lactation.

e Differences refer to values on day 110 of gestation vs. day 19 of lactation.

Initial (70 ± 2 d of gestation) BF thickness was similar between groups; however, LGPHASE sows tended to have greater BF at day 110 ± 2 of gestation compared to CON sows (*P *= 0.098). The increase in BF from day 70 ± 2 to day 110 ± 2 of gestation was greater in the LGPHASE group (*P *= 0.020). By 19 ± 3 d of lactation, BF did not differ between treatments, although the LGPHASE sows tended to lose more BF during lactation (110 ± 2 to 19 ± 3 d; *P *= 0.095). However, the LGPHASE sows tended to have a greater overall BF gain across the study period compared to CON sows (*P *= 0.074). Daily feed intake during both gestation and lactation was not different between treatments.

### Sow reproductive performance

Farrowing duration was shorter in the LGPHASE sows compared to the CON sows (*P *= 0.028) (Table 3). Total pigs born did not differ between treatments, nor did the number of liveborn piglets. However, the number of stillborn piglets and stillbirth rate tended to be lower in LGPHASE sows than in CON sows (*P *= 0.090 and *P *= 0.099, respectively). Average piglet birth weight was similar between groups (*P *= 0.858), as was total litter birth weight (*P *= 0.877).

**Table 3.** Effects of late gestation phase feeding on sow reproductive performance in cycle I.

Item	Dietary treatment		SEM	*P*-value
	CON	LGPHASE		
**Sows, n**	35	35	…	…
**Average parity, n**	1.61	1.61	…	…
**Farrowing duration, min^a^**	360	290	22.24	0.028
**Total born, n**	16.47	16.50	0.87	0.980
**Liveborn, n**	14.67	15.17	0.73	0.592
**Stillborn, n**	1.18	0.60	0.31	0.090
**Stillborn, %**	6.79	3.36	1.89	0.099
**Piglet birth wt., kg**	1.50	1.51	0.05	0.858
**Total litter birth wt., kg**	23.12	23.00	0.94	0.877
**Pigs weaned, n**	12.31	13.39	0.49	0.062
**Piglet wean wt., kg^b^**	6.16	5.70	0.22	0.087
**Total litter wean wt., kg^b^**	74.96	72.89	2.58	0.572
**Pigs weaned, %^c^**	84.80	90.46	1.67	0.019
**Pre weaning mortality, %**	14.87	10.03	1.98	0.045
**Wean age, days**	19.19	18.90	0.30	0.491

a Farrowing duration adjusted for total born as a covariate.

b Piglet wean weight and total litter wean weight adjusted for number of pigs weaned as a covariate.

c Pigs weaned, % = (Live piglets post-fostering ÷ Liveborn piglets) × 100.

The LGPHASE group tended to wean lighter pigs than the CON group (*P *= 0.087). However, the LGPHASE group also tended to wean more pigs than CON group (*P *= 0.062), and the LGPHASE group had greater piglet pre weaning survivability than the CON group (*P *= 0.019). Weaning age did not differ between groups (*P *= 0.491).

### Subsequent reproductive performance

Within-sow reproductive performance across consecutive cycles is presented in Table 4. Only sows that completed both reproductive cycles (*n* = 21 CON; *n* = 28 LGPHASE) were included in this analysis; sows removed after cycle I are reported in the sow removal summary (Table 5). Farrowing duration was shorter in LGPHASE-fed sows compared with CON-fed sows in cycle I (*P *= 0.006) and cycle II (*P *= 0.018), with no differences across cycles within either treatment. The number of total born and liveborn pigs increased from cycle I to cycle II in LGPHASE-fed sows (*P *= 0.024 and *P *= 0.031, respectively), whereas no cycle-to-cycle differences were observed in CON-fed sows. In cycle II, LGPHASE-fed sows had greater total born (*P *= 0.041) and liveborn (*P *= 0.015) than CON-fed sows. Stillbirth rates were lower in LGPHASE-fed sows compared with CON-fed sows during cycle I (*P *= 0.039) but did not differ across cycles within either group.

**Table 4.** Within-sow reproductive performance across two consecutive cycles with consistent dietary treatment.^a^

Item	CON	LG-Phase	SEM	*P*-value^b^
**Number of sows, n^c^**	21	28	…	…
**Average parity, n**	2.1	2.1	…	…
**Cycle I farrowing duration, min**	373	286	31.18	0.006
**Cycle II farrowing duration, min**	353	275	32.16	0.018
**SEM**	22.27	23.20	…	…
***P—*value^d^**	0.443	0.695	…	…
**Cycle I total born, n**	16.54	16.57	1.25	0.838
**Cycle II total born n**	16.84	19.24	1.28	0.041
**SEM**	0.89	0.91	…	…
***P—*value**	0.977	0.024	…	…
**Cycle I liveborn, n**	14.75	15.64	1.07	0.409
**Cycle II liveborn, n**	15.00	17.70	1.09	0.015
**SEM**	0.77	0.78	…	…
***P—*value**	0.787	0.031	…	…
**Cycle I stillborn, %**	7.94	4.44	1.65	0.039
**Cycle II stillborn, %**	6.00	4.52	1.68	0.795
**SEM**	1.16	1.20	…	…
***P—*value**	0.258	0.534	…	…

a Data represent only sows that completed both cycle I and cycle II (*n* = 21 CON, *n* = 28 LG-Phase); Cycle I data in this table may differ from values in Table 3 because this analysis excludes sows that were removed or died before cycle II.

b *P*-values within a cycle compare treatments.

c Sows were re-enrolled on their assigned diet at d 70 during cycle II.

d *P*-values across cycles evaluate differences between cycle I and cycle II within the same treatment.

**Table 5.** Sow removal reasons by dietary treatment.^a^

Reason for removal	Dietary treatment	
	CON	LGPHASE
**Abortion**	1	…
**Age**	4	4
**Euthanized; retained pigs**	…	1
**Euthanized; uterine prolapse**	1	…
**High stillbirth rate**	3	…
**Lameness**	…	1
**Negative pregnancy**	1	…
**No heat**	4	1

a Average parity of removed sows was 2.3 in CON and 3.6 in LGPHASE.

### Sow blood markers: Cycle I

**Fig. 1.**
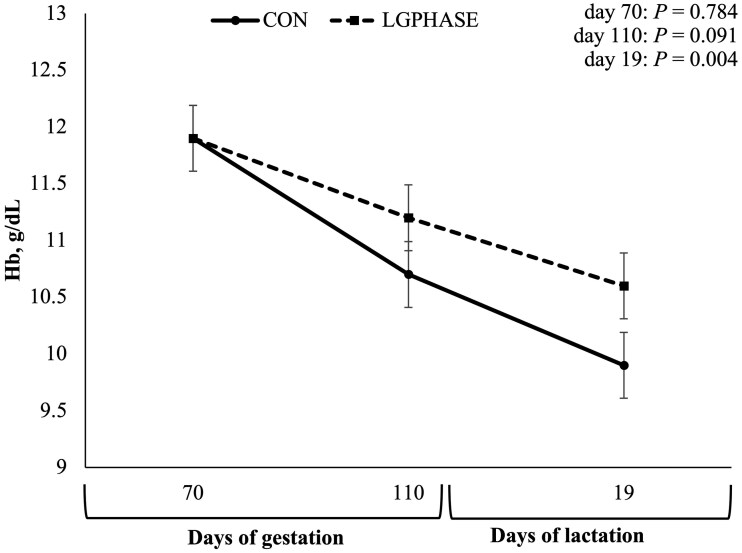
Effects of late gestation phase feeding on sow blood hemoglobin (Hb) levels during the first implementation cycle. Values represent means ± SEM; *n* = 35 sows per treatment (CON = 35; LGPHASE = 35).

Sow Hb concentrations (Fig. 1) did not differ by treatment group initially (70 ± 2 d of gestation); however, the LGPHASE group tended to have greater Hb concentrations at 110 ± 2 d of gestation (*P *= 0.091) and had greater Hb concentrations at 19 ± 3 d of lactation than the CON sows (*P *= 0.004). No differences were observed by treatment at any timepoint for RBC, WBC, PLT, or PCV (data not shown).

**Fig. 2.**
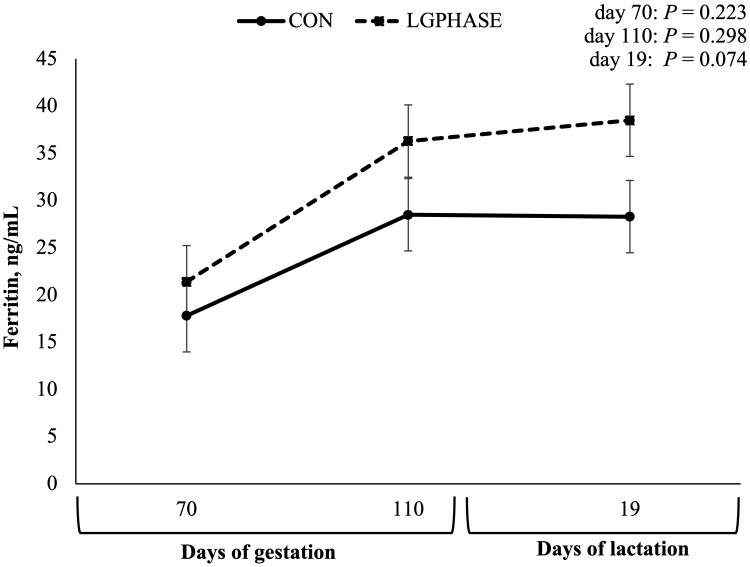
Effects of late gestation phase feeding on sow serum ferritin levels during the first implementation cycle. Values represent means ± SEM; *n* = 35 sows per treatment (CON = 35; LGPHASE = 35).

Sow serum ferritin levels (Fig. 2) did not differ between treatment groups initially (70 ± 2 d of gestation) or at 110 ± 2 d of gestation; however, the LGPHASE group tended to have greater ferritin levels at 19 ± 3 d of lactation compared to the CON sows (*P *= 0.074). Sow serum concentrations of copper, iron, and zinc did not differ at any timepoint between treatment groups (Table 6).

**Table 6.** Effects of late gestation phase feeding on sow serum trace minerals in cycle I.

Item, ppm	Dietary treatment		SEM	*P*-value
	CON	LGPHASE		
**Number of sows, n**	35	35	…	…
**Day 70**				
** Copper**	2.03	1.95	0.049	0.247
** Iron**	2.88	2.99	0.912	0.915
** Zinc**	0.52	0.47	0.026	0.145
**Day 110**				
** Copper**	1.80	1.91	0.054	0.115
** Iron**	2.69	2.85	0.402	0.715
** Zinc**	0.43	0.44	0.029	0.664
**Day 19**				
** Copper**	1.98	1.95	0.056	0.559
** Iron**	3.31	4.14	0.740	0.358
** Zinc**	0.68	0.73	0.032	0.294

**Fig. 3.**
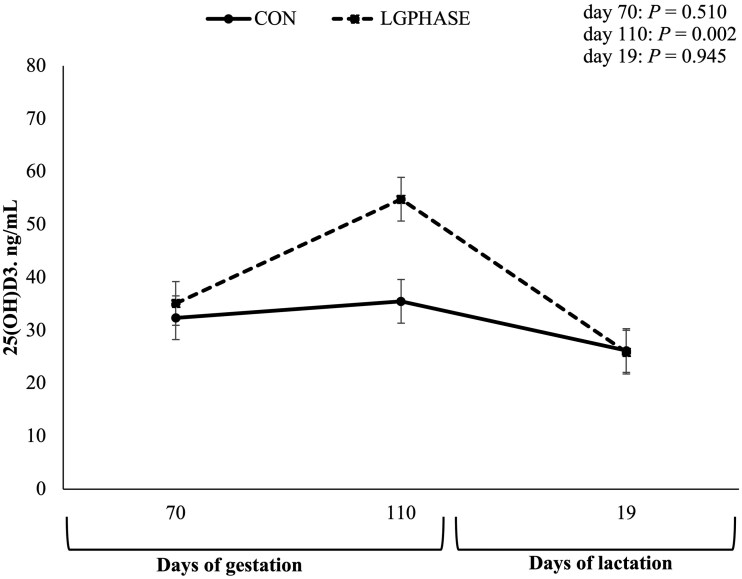
Effects of late gestation phase feeding on sow serum 25(OH)D_3_ levels during the first implementation cycle. Values represent means ± SEM; *n* = 35 sows per treatment (CON = 35; LGPHASE = 35).

Sow serum 25(OH)D_3_ levels (Fig. 3) did not differ between treatment groups initially (70 ± 2 d of gestation), however, the LGPHASE group had greater 25(OH)D_3_ levels at 110 ± 2 d of gestation than the CON group (*P *= 0.002). Sow serum 25(OH)D_3_ levels did not differ between treatment groups at 19 ± 3 d of lactation.

### Piglet blood markers: Cycle I

**Fig. 4.**
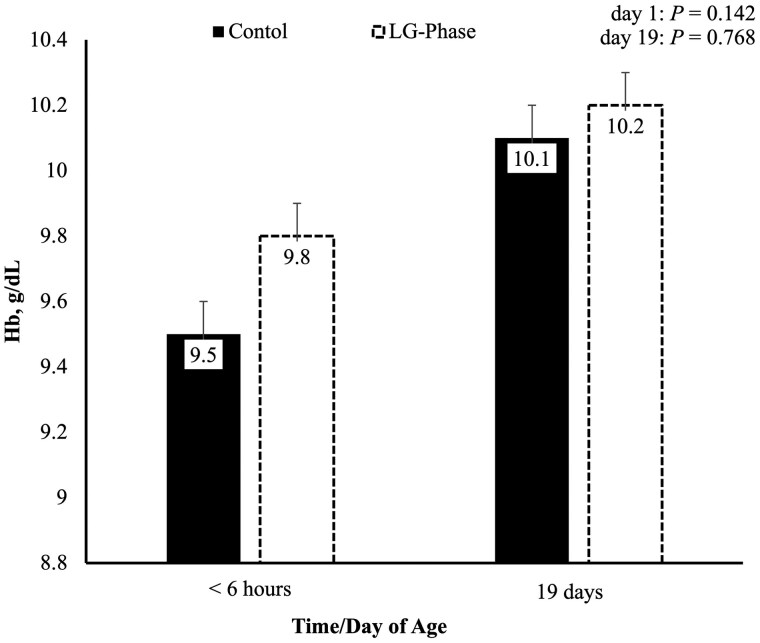
Effects of late gestation phase feeding on piglet blood hemoglobin levels during the first implementation cycle. Values are least squares means. Piglets sampled at birth: *n* = 974 (CON = 479; LGPHASE = 495). Piglets sampled at weaning: *n* = 826 (CON = 395; LGPHASE = 431).

**Fig. 5.**
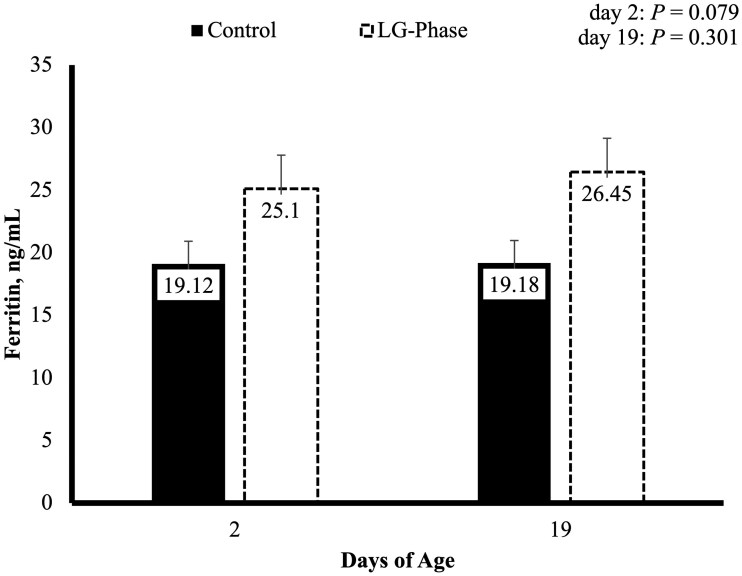
Effects of late gestation phase feeding on piglet serum ferritin levels during the first implementation cycle. Values represent means ± SEM; *n* = 70 pigs per treatment (CON = 70; LGPHASE = 70).

Piglet blood Hb (Fig. 4) did not differ at any timepoint. Piglet serum ferritin (Fig. 5) tended to be greater on day 1 of age in the LGPHASE group compared to CON (*P* = 0.079). Although serum ferritin did not differ statistically between treatments on day 19 ± 3.

Piglet serum concentrations of copper, iron, and zinc (Table 7) did not differ at birth (day 1 of age). Serum copper levels were greater in the LGPHASE group at weaning (19 ± 3 of age of age) than the CON pigs (*P *= 0.022), however, no differences in serum iron or zinc was observed at this timepoint.

**Table 7.** Effects of late gestation phase feeding on progeny serum trace minerals in cycle I.

Item, ppm	Dietary treatment		SEM	*P*-value
	CON	LGPHASE		
**Number of pigs, n**	140	140	…	…
**Day 1**				
** Copper**	0.55	0.55	0.022	0.964
** Iron**	4.80	5.13	0.579	0.679
** Zinc**	0.72	0.68	0.035	0.394
**Day 19**				
** Copper**	2.17	2.26	0.028	0.022
** Iron**	3.17	3.40	0.479	0.717
** Zinc**	0.62	0.67	0.099	0.698

**Fig. 6.**
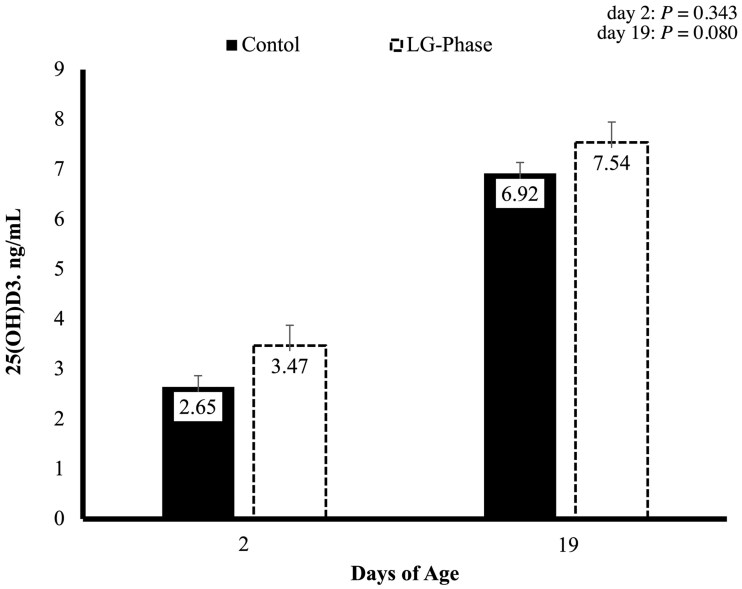
Effects of late gestation phase feeding on piglet serum 25(OH)D_3_ levels during the first implementation cycle. Values represent means ± SEM; *n* = 70 pigs per treatment (CON = 70; LGPHASE = 70).

Piglet 25(OH)D_3_ levels (Fig. 6) did not differ between treatment groups at birth (day 1 of age), however, 25(OH)D_3_ levels tended to be greater in the LGPHASE group at weaning (day 19 of age) than the CON group (*P *= 0.080).

## Discussion

This study was designed to determine whether increasing concentrations of amino acids, calcium, phosphorus, trace minerals, and vitamins in the diet during late gestation, when fetal growth and maternal demands peak, can improve reproductive performance, enhance maternal and progeny nutritional biomarkers, and support the sow through farrowing and postweaning recovery. The nutritional strategy aimed to better match nutrient supply with the increased nutrient demands of late gestation in prolific sows.

Reproductive performance metrics in cycle I, including total pigs born, number of liveborn piglets, and average piglet birth weight, did not differ between treatment groups. The absence of a treatment effect on total born was anticipated, as ovulation rate and embryonic survival are determined before the initiation of dietary treatments during late gestation (Geisert and Schmitt 2002). Improving piglet birth weight was not a primary goal of this intervention, so differences were not expected. Placental size and vascular capacity, which strongly influence birth weight (Town et al. 2004), are largely established earlier in gestation.

Sow BW in cycle I did not differ significantly between treatment groups at any single time point. However, changes observed during lactation suggest biologically meaningful differences: CON sows experienced a numerical decline in BW from farrowing to weaning. In contrast, LGPHASE sows had a modest numerical gain over the same period, indicating reduced reliance on body reserves. Such preservation of maternal tissue reserves is particularly important for sow recovery post-weaning and may enhance reproductive performance and longevity in subsequent cycles, where excessive tissue loss is often associated with delayed return to estrus and decreased conception rates (Cozannet et al. 2018).

In cycle I, LGPHASE sows tended to have greater pre-farrowing BF thickness than CON sows, likely reflecting the additional amino acid supply during late gestation that supported maternal protein and tissue accretion (Seoane et al. 2020). Greater amino acid supply likely allowed these sows to retain more protein and energy before farrowing, building maternal reserves for lactation. Insufficient fat reserves at farrowing have been linked to higher stillbirth rates, reduced piglet birth weight, and poorer lactational performance (Mun et al. 2024; Zhou et al. 2018). Despite higher pre-farrowing BF, LGPHASE sows lost more BF during lactation suggesting that improved nutrient reserves were mobilized to support milk production and litter growth. Notably, LGPHASE sows achieved a net gain in BF thickness over the full cycle, indicating that the enriched diet not only improved late-gestation nutrient status but also supported post-lactation recovery. Unlike excessive fat gain, which can suppress voluntary feed intake (Revell et al. 1998), modest BF increases in LGPHASE sows did not reduce lactation intake, suggesting improved nutrient status promoted metabolic flexibility, efficient energy utilization, and faster maternal recovery.

A key outcome in cycle I was a reduction in farrowing duration—an average improvement of 70 min—in LGPHASE sows, accompanied by a tendency for lower stillbirth rates. The relationship between prolonged farrowing, fetal hypoxia, and increased stillbirth risk is well established (Feyera et al. 2018; Langendijk and Plush 2019; Oliviero et al. 2010). The higher amino acid intake may have contributed to enhanced maternal protein status and hemoprotein synthesis, supporting uterine contractility and oxygen delivery to the fetus during parturition. The shorter farrowing duration observed in LGPHASE sows may be linked to improvements in maternal hematological status. Adequate maternal Hb is critical during parturition, supporting effective uterine contractility and minimizing fetal exposure to hypoxic stress (Kharate and Choudhari 2024; Zilliacus and Putkinen 1952). The improved Hb observed in LGPHASE sows at pre-farrow may have facilitated the farrowing process, as low maternal Hb concentrations have previously been associated with prolonged farrowing and increased stillbirth risk (McClellan et al. 2024a). Additionally, LGPHASE sows had higher circulating 25(OH)D_3_ concentrations pre-farrowing, consistent with the approximately twofold higher vitamin D content in the LGPHASE gestation diet. Vitamin D is critical not only for bone health but also for regulating calcium metabolism, which is essential for muscle function, including uterine contractility during labor (Holick 2007; Kovacs 2008).

Although serum ferritin concentrations did not differ between treatments at pre-farrowing in cycle I, LGPHASE sows tended to have higher ferritin levels at weaning and had significantly greater Hb, indicating improved maintenance or restoration of maternal iron stores and overall iron status during lactation (Chasteen and Harrison 1999). Late gestation and lactation are periods of elevated iron demand due to rapid fetal growth, elevated blood volume, and increased milk production (Mahan 2007; Matte and Girard 1996; Peters and Mahan 2008). The higher protein availability in LGPHASE sows likely supported synthesis of transferrin and other iron-binding proteins, improving iron transport and utilization in both sow and piglet. The improvements in ferritin and Hb observed at weaning in LGPHASE sows likely reflect the benefits of enhanced overall nutrient status entering the postpartum period. Iron metabolism depends on several key nutrients beyond iron itself, including protein (for transferrin synthesis), copper (involved in ferroxidase activity), vitamin B6 (a cofactor in heme synthesis), and vitamin C (which enhances intestinal iron absorption and maintains iron in its reduced, bioavailable form) (Andrews 1999; Hallberg et al. 1989; Linder and Hazegh-Azam 1996; Schulman and Richert 1957; Underwood and Suttle 1999). The multi-nutrient supplementation strategy employed in this study, rather than iron supplementation alone, may have contributed to the observed improvements in maternal iron status.

Both dietary treatments contained exclusively organic trace minerals; however, the LGPHASE diet provided these minerals at higher levels. Previous research has demonstrated benefits of replacing inorganic trace minerals with organic forms, particularly for improving progeny mineral status and reproductive performance (McClellan et al. 2025; Wang et al. 2014). The results of cycle I suggest that both the source and the concentration of organic trace minerals are important, as the elevated levels in the LGPHASE diet were associated with improved maternal iron status and enhanced iron transfer to offspring. This enhanced maternal mineral status likely supported better replenishment of iron stores during lactation, as reflected by higher ferritin levels and Hb concentrations at weaning. This response likely reflects not only the higher trace mineral supply but also the contribution of higher amino acid availability and vitamins in supporting mineral-binding proteins and overall metabolic efficiency. Together, these factors may help sows better meet the heightened demands of late gestation and lactation.

While piglet Hb concentrations in cycle I did not differ between groups at birth, piglets from LGPHASE sows tended to have higher serum ferritin levels, suggesting improved transplacental iron delivery. Neonatal pigs are born with limited hepatic iron stores and undergo rapid erythropoiesis postpartum, making them particularly vulnerable to iron deficiency (Szudzik et al. 2018). Although all piglets in this study received an iron injection by day two of age, the period between birth and supplementation represents a critical window during which adequate iron reserves are essential to support early oxygen transport and metabolic demands (McGowan and Crichton 1923; Ullrey et al. 1953).

At birth, piglet serum concentrations of iron, copper, zinc, and vitamin D did not differ between treatments, likely reflecting maternal physiological constraints on fetal micronutrient accumulation during gestation. In pigs, the epitheliochorial placenta limits passive mineral transfer, requiring specialized mechanisms, such as uteroferrin-mediated iron transport to regulate fetal mineral supply (Ducsay et al. 1986). This placental regulation, combined with uterine crowding in large litters, may further restrict individual access to maternal nutrients (Madsen et al. 2023).

Although no differences were observed at birth, by weaning in cycle I piglets from LGPHASE sows had higher serum copper concentrations and tended to have greater 25(OH)D_3_ levels by weaning in cycle I. While colostrum and milk composition were not measured, the maternal diet prior to farrowing may have influenced milk nutrient content. Additionally, the improved neonatal iron status observed in LGPHASE piglets may have supported gut development and epithelial integrity, potentially enhancing absorption of other micronutrients during the suckling period (Zhou et al. 2020). Both copper and vitamin D are critical for neonatal growth and health: copper is essential for oxidative defense, connective tissue development, and immune cell function (Forouzandeh et al. 2022), whereas vitamin D supports calcium metabolism, bone formation, immune modulation, and maintenance of intestinal barrier integrity (Assa et al. 2014; Baeke et al. 2010; Kong et al. 2008; Lips and van Schoor 2011; Madsen et al. 2023).

These improvements in piglet micronutrient status were reflected in early-life outcomes. The LGPHASE sows weaned more piglets because their litters had higher pre-weaning survivability than CON sows, likely reflecting the combined benefits of enhanced maternal nutrient status and greater neonatal resilience. Although individual piglet weaning weight tended to be lower in the LGPHASE group, a common trade-off in larger litters due to increased competition for milk (Andersen et al. 2011; Mata et al. 2025), total litter weaning weight did not differ between treatments, indicating that overall litter productivity was maintained despite the higher number of offspring. Maternal amino acid intake may have helped support milk protein synthesis, buffering the litter against the potential negative effects of increased litter size.

The positive effects of the LGPHASE diet extended beyond reproductive performance, contributing to greater sow longevity and fewer removals in the subsequent cycle. The LGPHASE-fed sows had numerically fewer removals (*n* = 7) compared with CON sows (*n* = 14). Although inferential statistics were not applied due to the limited sample size, this disparity suggests that enhanced late-gestation nutrition supported maternal resilience, reproductive longevity, and overall herd sustainability. Most removals in CON sows were due to reproductive failure, whereas removals among LGPHASE sows were fewer and more evenly distributed across causes.

Analysis of within-sow reproductive outcomes across consecutive cycles demonstrated consistent improvements in LGPHASE sows. Farrowing duration remained shorter in both cycles, and total and liveborn piglets increased from Cycle I to Cycle II in both treatment groups. Stillbirth rates were lower in LGPHASE sows compared with CON-fed sows in Cycle I. During Cycle II, the study farm experienced a confirmed porcine circovirus 3 challenge, which is known to increase stillbirth incidence (Tochetto et al. 2020) and may have masked treatment differences in that cycle. Overall, these findings highlight the potential for late-gestation nutrient enrichment to support prolificacy across multiple parities. A potential limitation of this analysis is the within-sow design, which introduces the possibility that parity progression influenced outcomes; however, average parity was similar between groups, and trends remained consistent across cycles, supporting the robustness of the results.

Collectively, the findings from this study emphasize the importance of treating late gestation as a nutritionally distinct phase in the reproductive cycle. This study provides empirical evidence that enhancing amino acid, mineral, and vitamin intake simultaneously—rather than addressing each nutrient in isolation—can effectively meet the elevated nutrient demands of prolific sows in late gestation resulting in improved hematological status, reproductive outcomes, micronutrient transfer, piglet survival, and potentially sow retention. Evaluating the subsequent reproductive cycle revealed that these nutritional benefits can translate into increased performance in the following parity, including improved litter size and maternal recovery. These results also highlight the value of monitoring sow-specific outcomes, such as hematological status, nutrient biomarkers, and recovery indicators, to better capture the full benefits of nutritional interventions beyond piglet performance alone. Future research should refine individual nutrient requirements and develop practical strategies for implementing phase-feeding programs in commercial production systems.

## Disclosures

J. Acosta and B. Lawrence are employed by Novus International, Inc.; S. Hough and J. Bergstrom are employed by dsm-firmenich; and M. Weaver and R. Robbins are employed by Pig Improvement Company. Their roles were limited to the review of the manuscript.

## List of abbreviations:

AOAC: Association of Official Analytical Chemists
BF: Backfat
BW: Body weight
Hb: Hemoglobin
PCV: Packed cell volume
PLT: Platelet
RBC: Red blood cell
SID: Standardized Ileal Digestible
25(OH)D_3_: 25 hydroxyvitamin D
WBC: White blood cell
